# Semantic science and its communication - a personal view

**DOI:** 10.1186/1758-2946-3-48

**Published:** 2011-10-14

**Authors:** Peter Murray-Rust

**Affiliations:** 1 Unilever Centre for Molecular Science Informatics, Department of Chemistry, Lensfield Road, Cambridge CB2 1EW, UK

## Abstract

The articles in this special issue represent the culmination of about 15 years working with the potential of the web to support chemical and related subjects. The selection of papers arises from a symposium held in January 2011 ('Visions of a Semantic Molecular Future') which gave me an opportunity to invite many people who shared the same vision. I have asked them to contribute their papers and most have been able to do so. They cover a wide range of content, approaches and styles and apart from the selection of the speakers (and hence the authors) I have not exercised any control over the content.

## Overview

The articles have a common theme of representing information in a semantic manner - *i.e*. being largely "understandable" by machine. This theme is common across science and many of the articles can and should be read by people outside the chemical sciences, including information scientists, librarians, *etc*. An emergent phenomenon of the last two decades is that information systems can grow without top-down directions. This is disruptive in that it empowers anyone with energy and web-skills, and is most powerful when exercised in communities of people with similar or complementary skills.

It is often possible to move very quickly, and in our hackfests (one was prepended to the symposium) we have shown that it is possible to prototype within a day or two. This creates a new generation of scientist-hackers (I use "hacker" as "A person who enjoys exploring the details of programmable systems and stretching their capabilities" [1]). Several of the authors in this issue would regard themselves as "hackers" and enjoy communicating through software and systems rather than written English. This stretches the boundaries of the possible but also creates tension where the mainstream world cannot react on a hacker timescale and with hacker ethics.

More generally many scientists and information professionals are increasingly frustrated with the conventional means of disseminating science. Most conventional publishers regard scientific articles as "their content" and a very recent article (2011-06-20) from the STM publishers [2] indicates that the publishers believe they have the right to determine how content is, or more often is not, used. As an example most forbid by default indexing, textmining, repurposing, even of factual data to which the scientist has a legitimate subscription. This has an entirely negative effect on information-driven science, preventing even the development of the technology.

Generally, therefore, there is a culture of bottom-up change ("web democracy") which looks to the modern web and examples of empowerment. (There are also examples of disempowerment such as attacks on Net-neutrality, walled gardens, information monopolies, vendor lock-in, *etc*. and this contrast activates many in the modern informatics world). There are several articles, therefore, whose main theme is the access to Open information.

## Openness and the choice of BMC as publisher

I have been critical of many publishers for their stance on closed information, and resolved that the issue reporting the symposium had to be completely Open. This is difficult in chemistry where there are almost no "Open Access" journals (those where by default all articles are Open ("Gold")). The "Green" approach, where articles may be posted free-as-in-beer but not free-as-in-speech (*e.g*. CC-BY), is useless in science as it is impossible to discover and harvest green articles. Hybrid journals (where articles may be made Open by publication charges) are also of little value as the rights to the contents are usually poorly labelled and a machine cannot discover all "Open chemistry articles".

While writing this overview and several articles I have become even more convinced that the only way of creating full semantic science is to publish Openly (CC-BY) and to publish completely (*i.e*. all experimental information (CC0/PDDL)). I believe that most funders now recognise this and are pushing, as hard as they can, to create fully Openly published science. I think this has to come, the question is how long it takes and in what form.

I now believe that in many cases it is unethical to restrict access to publicly funded science. Lessig, in his CERN talk ("Scientific Knowledge Should Not Be Reserved For Academic Elite" [3]), showed that it would cost 500 USD for him to read the top 10 papers relating to his child's condition. These papers are effectively only available to academics in rich universities. A colleague recently told me he had spent a month researching the literature of his child's condition (to critically effective purpose) and we agreed he could only do this because he was a professor at a University. That is one reason I support the Open Knowledge Foundation and its projects to define and obtain Open information (of which Open Bibliography [4] in this issue is typical).

As part of this effort four of us (including authors in this issue) developed the Panton Principles for Open Scientific Data. These principles are simple and, we hope, self-evidently worth pursuing and would lead to a greatly increased substrate for the Scientific Semantic Web. We were therefore delighted when BioMed Central not only enthusiastically adopted the idea but took positive steps to implement this as part of their publication process, for example by labelling data items with the OKF's "OPEN DATA" logo. This is valuable not only in making the data repurposable, but also by promoting the concept - many readers will now be familiar with the logo. BMC have also encouraged authors (and editors) to highlight outstanding examples of data publication (and done me the honour of asking me to present their awards).

It is therefore a real pleasure to work with a publisher who understands my, and my co-authors', intentions and is prepared to work to make them happen. The article explores many new types of publications and BMC have undertaken, as far as technically possible, to implement them as examples of a new generation of publication technologies. I and others have been critical of PDF as a publication format - it destroys semantics and innovation, but we must "eat our own dogfood" [5] and this is shown by several articles. Henry Rzepa creates all his molecules as semantic objects, while in Open Bibliography [4] we use our newly developed BibJSON and ScholarlyHTML to create and publish the article.

I am confident that because of the Openness, the readership of these articles will be much larger than if they were published in a closed access manner, however apparent the prestige of the closed access publisher. It is easy for a mature scientist, such as myself, to publish in an Open Access journal as it is unlikely to affect my career. I'd like to pay credit to all young people who have decided to publish in OA journals despite the possible current (irrational) view that this is detrimental to how they are regarded. I believe that their faith will be justified and that in a very short time the work published here will have higher visibility, and possibly regard, than if it had been published in an apparently more prestigious, closed access journal.

## Open Data

Five years ago the term "Open Data" was unknown (I started a Wikipedia page [6] to collect instances of usage). Now it is ubiquitous. Most of the public funders (Research Councils UK, Wellcome Trust, NIH, NSF and other national bodies) are now requiring that researchers make their data Openly available.

The first challenge is cultural; researchers have to be persuaded that Open Data is not only inevitable but also beneficial to their activities. Even when an author is convinced of the value of publishing Open Data, it is usually not trivial to do so. Unlike a manuscript where a static, human-readable, webpage can be posted and served for all time, data are frequently much more complex. They may be very large (petabytes), complex in both semantics and organisation, and even distributed over several sites. In bioscience, it is becoming commoner to see data published as Excel and other spreadsheets but in chemistry (apart from crystallography) the tradition is still to publish supplemental data as PDF, which destroys much of its semantics. One simple and achievable goal of these publications is to convince chemists that publishing in semantic form is "almost" no effort, compared to the effort of producing the data in the first place. If we were able to persuade researchers in computational chemistry simply to deposit their logfiles (usually less than 5 MB), or the Word documents for their syntheses, machines would be able to revolutionise the practice and understanding of computational and experimental chemistry. Open Access (CC-BY) implies (but may not explicitly state) that articles can be repurposed by machine extraction of data items, *e.g*. by OSCAR.

We have also addressed the question of what is Open Data and how do we identify it, both to humans and to machines. For many chemists, this may be the first time that they have had to consider this problem, but it is becoming increasingly required in many fields and for that reason, we have in several papers, discussed the question of licenses and contracts.

## The semantic vision

I was excited and entranced by chemical informatics in the mid-70s as a result of some of the ground-breaking work done between chemists and computer scientists. The visions of LHASA, CONGEN, DENDRAL and others opened up the prospect of a chemical world where machines were seen as valuable allies of humans. This vision was also held in the world of chess, and indeed many chemical informatics processes are similar to the operations required in 'artificial intelligence'. Chess has succeeded. Machines can now beat any human on the planet. For whatever reasons, chemistry turned its back on AI and there have been few developments in the last three decades. A necessary condition is the Open availability of semantic data, and if this comes about then there will be a major discontinuity in the way we practice chemistry.

In 1994, Henry Rzepa and I attended the first WWW conference in CERN. It was a remarkable occasion where a number of very early adopters showed what was possible with web technology and gave a vision of how this would change the way that science was not only reported but also done. There was a feeling that we were entering a new frontier where anything was possible and where new rules would evolve to fit the vision of cyberspace. The final session, where Tim Berners-Lee showed how semantic operations altered the real world was one of the seminal events of my last 20 years.

## Semantic reality

Not surprisingly, semantic progress has turned out very differently from our original visions. We have stuck to our view that science must adopt semantic technologies including both the formal description of objects and the links between them. Chemistry has been very slow to adopt this, but other subjects have been much more adventurous and in bio- and geo-sciences it is routine to create objects which are derived from, and linked to, other objects.

Many of the problems are cultural and for that reason several of the papers in this issue address the need to change attitudes as much as the technical requirements for the electronic infrastructure. I believe that it is impossible to do modern science unless the key information is completely Open. This applies, for example, to identifier systems, bibliographic data and much factual data. Chemistry, unfortunately in my opinion, has a strong ingrained culture of possession and sale/licensing of data. For this reason, it is often behind other subjects and, in the recent SOAP report [7] chemistry was highlighted as several years behind bioscience in its approach to Openness.

For that reason, some of the things we report are prototypes rather than completely established semantic resources. The biosciences have convinced funders that it is valuable to have completely Open access to sequences, structures, ontologies, *etc*. In chemistry, most of the freely accessible material has been produced by enthusiasts rather than large funded organisations. Indeed, it is the availability of bioscience resources such as ChEBI which to some extent drive the adoption of Open chemical semantics.

It is also an opportunity for our group to summarise formally several of the projects that they have been working on for several years. It is a feature of information projects that there is often no clear point at which a formal publication is immediately relevant and indeed this highlights the disconnect between publishing necessary information and publishing to acquire a community seal of approval ('a publication').

## Chemistry as a community

Many disciplines have a close sense of community (I highlight crystallography which has a real sense of communal practice and goals). Many of the ideas in these articles have been inspired by crystallographic practice, its outstanding scientists, and its International Union - probably the leader in driving semantic approaches.

Scientific communities are now common on the web (and even have commercial value) and several of the articles emphasise the role of *ad hoc *and other communities. The web has the great advantage that anyone can, relatively easily, find those people and organizations who share values and goals, amplifying minority or early-adopter initiatives. Their dynamics are unpredictable and most die, but enough survive to provide world-changing mechanisms.

There is no clear community focus for chemistry overall (though sub sections - such as WATOC (World Association of Theoretical Organic Chemists) may provide one). The main drivers (funding, advancement, commerce) have always been present but the modern era has amplified and often dehumanised them. With growing emphasis on publication to generate the income of learned societies there is a decreasing sense that they act as nuclei for community to grow communal goods.

Because of this, chemistry has almost no public ontologies, and we have a vicious circle. Without ontologies, authors cannot reasonably be expected to create semantic information, and without a clear need for semantic information, the community will not take on the considerable load of creating ontologies. Several of the articles argue that the creation of lightweight dictionaries and other semantic metadata is affordable by the community and I believe that if the communal will is present, then it would be possible through bodies such as IUPAC and others, to create a full semantic infrastructure for much of the current published chemistry.

The current legal and contractual restrictions on re-using chemical data are seriously holding chemistry behind other subjects. These articles in this issue are not the place for polemics but we hope that traditional creators of information resources in chemistry will now think carefully about the value of making their data fully Openly available. This will be a considerable act of faith, because it will need a change in business model. Some of those providers have been traditionally held in high esteem by the community and if they use that esteem they have the opportunity to change the practice of chemical informatics.

## The value of informatics

A major feature underlying all of the papers is to give an insight into the process of creating an information ecology. Some of them represent scientific discoveries (*e.g*. Rzepa) but most are concerned with building a coherent infrastructure usable by the community. It may be useful to liken this infrastructure to the development of instrumentation in many branches of science. Science depended on the microscope, the telescope, the spectrograph, the Geiger counter and many other types of instrumentation. There is sometimes a modern tendency to discount instrumentation and infrastructure as not being 'proper science'. We hope that this issue will redress that balance.

As an analogy, Mendeleev required access to other scientists' work to produce his classification, as did Pauling, Woodward and Hoffmann. I believe that the current chemical and related literature contains considerable amounts of undiscovered science, and that with 'information telescopes' we can start to discover this.

The development of infrastructure is a lengthy process. The web has, perhaps, given us an optimistic idea of the speed at which new ways of working can be implemented. We are still often governed by Planck's observation ("Science progresses one funeral at a time") and this is equally true for some areas of informatics. Several of the articles reflect the difficulty of catalysing change in what is essentially a mature and therefore conservative discipline.

Henry Rzepa and I were active contributors to the development of XML by running the XML-DEV mailing list (1997). This was a highly successful Open example of true collaboration and for me it culminated in the development of the SAX protocol late that year. XML had been seen as a primarily document- plus typesetting-oriented discipline, but some of us realised its potential for data modelling and transfer, and therefore the need for APIs in XML tools. I nagged continually at the community, and, as a result, Tim Bray, David Megginson and others helped us to develop the SAX protocol, now implemented in every computer on the planet. This protocol was developed in a calendar month and has stood the test of time exceedingly well.

This, perhaps, gave Henry, myself and other early adopters a false vision of how rapidly we would be able to take these new ideas to chemistry. Over the decade 2000-2010, we have developed and published specifications and software which we believe represent a formal but implementable infrastructure for chemical informatics. The uptake of these has been slow, but unlike some new technologies has not gone through the hype and depression syndrome (Gartner curve). In fact, this timescale is not so unusual. HTML itself has been through nearly 20 years of deployment and only now, with HTML5, does it appear that the community is starting to work together rather than fracturing for organisational and personal advantage. Similarly, semantic MathML is taking many years to become established. It is not that these systems, including CML, have been supplanted by 'better' ways of doing things, but more that the community as a whole is yet to be enlightened about the value of semantics.

## Publishing

Scientific publishing should be a key part of the semantic revolution, but it has so far completely failed to address the vision. This is ironic in that HTML, which catalysed the web, was developed as a way for scientists at CERN to share information, but we have currently regressed to a completely non-semantic (PDF) manner of communication. This has replicated the traditional paper format so well that the only discernable value is to transfer the printing bill from the publishers to the readers. Not only has this held back our imagination, but has actually moulded the new, and I think somewhat unfortunate, values in the publication process. In many cases, authors now publish primarily to attain numerical estimates of worth above communication, validating experiments and other fundamental aspects of the process.

The web can, and, we hope, will, change this. Where you publish should not matter so long as the material is discoverable and the process of reviewing is understood. I believe that the papers in this issue will be read well beyond the cheminformatics community, because their value will be discerned and communicated by methods supplementary to the formal publishing process.

A major challenge in this issue is that the timescales for many of the projects is complex. In many lab experiments (such as chemical synthesis or chemical crystallography) the process is clearly bounded. "make this compound", "check success through crystal structure analysis". Each (normally) has a clear endpoint and can be published as a static document.

In contrast how should we publish software? We use public repositories and these contain a complete record and the current semantic object. If we wish to tell the world about a development we put it on the mailing list. There is no need for a formal publication for those aspects. The motivation is therefore primarily to establish our reputation and there is no simple way to decide when this should be done. JUMBO has had six revisions - should this result in six papers or one or none? (Actually the only JUMBO paper is in 1997 [8]). Six papers would confuse - but after 14 active years it's time for another, I think, which explains the design process. OSCAR3 has its citable publication - a few years back - and we feel it's useful to publish our current ideas, which have more to do with software engineering than new chemical entity recognition.

Or data? Crystaleye was a spinoff from Nick Day's thesis - it wasn't planned as a separate project - but simply a knowledgebase to use for his calculations. It does not have a formal publication other an archive of a presentation [9]. The system has been running 5 years without serious mishap but the lack of a formal publication makes it difficult to write papers which refer to it. So we shall do this - after the fact. But if we had a semantic publication process it would be "published" by now.

## The need to change publication processes

Historically the scientific community has required the following from the publication process:

• Establishment or priority and authorship

• Exposure and preservation of the scientific record

• Communicating the science to one's peers and the wider world

• Allowing the science to be moderated by peers and others ("reviewing").

There is perhaps an additional axis in today's bibliometric-obsessed world: allowing the work to receive an official assessment of merit.

However the publication process is out of sync with the modern web-based world ("Web 2.0") which allows the publication process to encourage and support:

**• Collaborative working **(as seen in many projects such as Wikipedia, Open StreetMap, and in science, Galaxy Zoo). Here each contribution is often an atom in a much larger cloud and the publication process is continuous rather than discrete. Wikipedia articles are "never finished" though there are some efforts to provide frozen versions. This is a strong theme of this "issue".

**• Independence of the source of publication**. Given the ability of search engines, and the social networks, to discover anything of value it matters less *where *something is published. Other than the choice of reviewers the primary issues is whether a piece of information is accessible or limited. History has shown that high quality scholarship on the web will usually surface regardless of where it is published.

**• Creation of continuous semantic objects**. By recording everything we do, annotating it, and revising it, we can maintain a current semantic publication object at all times, including a revisitable history. This should be the object of scientific publication, not the current PDF.

**• The paper (semantic object) as a driver of research**. The idea of writing a paper before the research is carried out is valuable and not novel (*e.g*. George M. Whitesides [10,11]). Here, however, we extend the paper to semantic objects (programs, spreadsheets, molecules, bibliography, *etc*.).

Several of the papers in the article have adopted these later ideas. This has been most obvious in Open Bibliography [4] where effectively the whole concept and technology has been driven during the 6 weeks of "writing the paper". We started with a blank page and four people (William Waites, Mark MacGillivray, Ben O'Steen, Peter Murray-Rust) and during the writing process brought in new authors (Jim Pitman, Peter Sefton, Richard Jones) and communally created the design, technology and "paper". The introduction of Scholarly HTML made this paper self-referential. The Quixote paper [12] has also dramatically driven the design of Quixote, particularly the social aspects.

## The content of the issue

Several of the articles (CML [13], OSCAR [14], OPSIN, dictionaries [15], WWMM [16]) in this issue cover a decade of work. We hope this will be useful to scientists and scholars who wish to implement new ideas and to give them some idea of what works, and what, more commonly, does not work. Sometimes only the passage of time and persistence achieves some level of success. Again, the short-termism of many infrastructural projects militates against developing a good platform for the future.

The long timescales highlight the difficulty of conventional publication. The world knows of these projects through blogs, online resources, user communities and so on, and a conventional learned paper has little value in communicating or preserving. Its prime merit is to achieve a traditional numeric merit for the work, often delayed by several years through the citation mechanism. I believe that it is important to change the values that we use in our assessment of on-going scientific endeavours, and avoid ritual publication.

Some of the articles (Wilbanks [17], Neylon [18]) discuss the philosophy and practice of new models of scientific endeavour and communications. Some of the articles have a retrospective look (CML [13], Zaharevitz [19]) but the fundamental principles are still as important today as when the work was started. A number represent growing points whose development is highly unpredictable. These include the WWMM [16], where the vision of a distributed peer-to-peer knowledge resource has had to wait a decade until it could be implemented. The Quixote project is only months old but takes this vision and has already built an impressive prototype, which I expect to set the model for computationally-based knowledge repositories. These projects rely heavily on community, and this is most clearly shown in the Blue Obelisk movement [20] which aims to, and has largely succeeded in, creating an Open infrastructure for cheminformatics. A major motivation for this has been not just that software and data should be universally available but also that this is the only manner in which science can be reputably validated both by humans and machines. An example of the need for such validation is shown in Henry Rzepa's article [21].

The OpenBibliography project represents a socio-political imperative whose time has come, and for which the technology is appropriate. A year ago the JISC-funded OpenBibliography project could not point to a significant amount of open resources, but in the last year we have helped to catalyse the release of both library data (BL, CUL and several others), and also of scientific bibliography. It is impossible to find Open resources for scientific bibliography but we believe that in a year's time, readers can look back and see this as a key starting point. It is worth noting that the very process of writing this article has generated a great deal of new formalism and tools in Open bibliography, and effectively given major impetus to the BibJSON approach.

Other articles (OSCAR, Open patents [22], dictionaries, CML and CMLLite [23]) describe the design and implementation of information systems. In general, there is little funding for developing scientific software, though we have been fortunate to receive some from eScience and from JISC. We have taken this responsibility very seriously and our group has installed many of the cutting-edge ideas and tools for building high-quality systems. Members of the group collaborate and use common servers for their work (as far as possible on Open sites). Software libraries are used and re-used between group members, and we have developed a culture of communal ownership and responsibility. By using the continuous integration system (Jenkins), a failure in one library can immediately be highlighted and corrected before it impacts on other projects. Where funding is available, and where the culture allows it, we would very strongly recommend these practices in other groups. Again, many of these systems have taken over a decade to evolve from initial concepts to mature libraries, but we believe that almost all the systems reported in this article have been heavily re-factored and, within the academic environment, represent an attainable level of quality.

## The future

Several articles are growing points, perhaps none more than AMI [24] where we explore the human-cyber interface in a laboratory, a "memex" which may ultimately replace some (but hopefully not all) of the role of the chemistry laboratory. In the same way Quixote represents a memex for computational chemistry. There is no clear pathway for AMI (and I predict that this will be largely influenced by what happens in the domestic arena).

The relative stagnation of chemical informatics suggests that change is unlikely to happen from within chemistry. As progress occurs in other areas (retail, bioscience *etc*.) chemistry may be dragged into the semantic world regardless. If chemists wish to retain control over their own systems they will be wise to start investing in Open semantic environments, because otherwise the rest of the world will do it for them.

How can chemical informatics survive and prosper? I think the most likely model will be Open publishing, not just of texts but data and other resources, mandated and paid for by funders. Those publishers which are able to adopt an Open model rather than continuing to maintain their own walled gardens, will ultimately triumph, and probably more rapidly than we expect.

## References

[B1] Wikipedia: Hacking (innovation) http://en.wikipedia.org/wiki/Hacker_%28programmer_subculture%29

[B2] Smit E van der Graaf M Journal Article Mining: A Research study into Practices, Policies, Plans... and Promises Publishers Research Council 2011 153

[B3] Intellectual Property Watch: Lessig At CERN: Scientific Knowledge Should Not Be Reserved For Academic Elite http://www.ip-watch.org/weblog/2011/04/19/lessig-at-cern-scientific-knowledge-should-not-be-reserved-for-academic-elite/

[B4] Jones R MacGillivray M Murray-Rust P Pitman J Sefton P O'Steen B Waites W Open Bibliography for Science, Technology, and Medicine J Cheminform 2011 3 47 10.1186/1758-2946-3-47 PMC3206455 21999661

[B5] Wikipedia: Eating your own dog food http://en.wikipedia.org/wiki/Eating_your_own_dog_food

[B6] Wikipedia: Open science data http://en.wikipedia.org/wiki/Open_science_data

[B7] Study of Open Access Publishing: Report from the SOAP Symposium http://project-soap.eu/report-from-the-soap-symposium/

[B8] Murray-Rust P JUMBO: An Object-based XML Browser World Wide Web Journal 1997 2 4 197 206

[B9] Downing OJ Day Nicholas E Murray-Rust P CrystalEye - From Desktop to Data Repository http://www.dspace.cam.ac.uk/handle/1810/196186

[B10] Wikipedia: George M. Whitesides http://en.wikipedia.org/wiki/George_M._Whitesides

[B11] Whitesides GM Whitesides' Group: Writing a Paper Advanced Materials 2004 16 1375 1377 10.1002/adma.200400767

[B12] Adams SE de Castro P Echenique P Estrada J Hanwell MD Murray-Rust P Sherwood P Thomas J Townsend J The Quixote project: Collaborative and Open Quantum Chemistry data management in the Internet age J Cheminform 2011 3 38 10.1186/1758-2946-3-S1-P38 PMC3206452 21999363

[B13] Murray-Rust P Rzepa HS CML: Evolution and Design J Cheminform 2011 3 44 10.1186/1758-2946-3-44 PMC3205047 21999549

[B14] Jessop DM Adams SE Willighagen EL Hawizy L Murray-Rust P OSCAR4: a flexible architecture for chemical text-mining J Cheminform 2011 3 41 10.1186/1758-2946-3-S1-P41 PMC3205045 21999457

[B15] Murray-Rust P Townsend J Adams SE Phadungsukanan W Thomas J The semantics of Chemical Markup Language (CML): dictionaries and conventions J Cheminform 2011 3 43 10.1186/1758-2946-3-S1-P43 PMC3206453 21999509

[B16] Murray-Rust P Adams SE Downing J Townsend J Zhang YY The semantic architecture of the World-Wide Molecular Matrix (WWMM) J Cheminform 2011 3 42 10.1186/1758-2946-3-S1-P42 PMC3205046 21999475

[B17] Wilbanks J Openness as Infrastructure J Cheminform 2011 3 36 10.1186/1758-2946-3-S1-P36 PMC3197551 21999327

[B18] Neylon C Three stories about the conduct of science: Past, future, and present J Cheminform 2011 3 35 10.1186/1758-2946-3-S1-P35 PMC3198952 21999290

[B19] Zaharevitz DW Adventures in Public Data J Cheminform 2011 3 34 10.1186/1758-2946-3-S1-P34 PMC3198951 22017861

[B20] O'Boyle N Guha R Willighagen EL Adams SE Alvarsson J Bradley JC Filippov IV Hanson RM Hanwell MD Hutchison GR James CA Jeliazkova N Lang ASID Langner KM Lonie DC Lowe DM Pansanel J Pavlov D Spjuth O Steinbeck C Tenderholt AL Theisen KJ Murray-Rust P Open Data, Open Source and Open Standards in chemistry: The Blue Obelisk five years on J Cheminform 2011 3 37 10.1186/1758-2946-3-S1-P37 PMC3205042 21999342

[B21] Rzepa HS The past, present and future of Scientific discourse J Cheminform 2011 3 46 10.1186/1758-2946-3-46 PMC3208583 21999632

[B22] Jessop DM Adams SE Murray-Rust P Mining chemical information from Open patent J Cheminform 2011 3 40 10.1186/1758-2946-3-S1-P40 PMC3205044 21999425

[B23] Townsend J Murray-Rust P CMLLite: a design philosophy for CML J Cheminform 2011 3 39 10.1186/1758-2946-3-S1-P39 PMC3205043 21999395

[B24] Brooks BJ Thorn AL Smith M Matthews P Chen S O'Steen B Adams SE Townsend JA Murray-Rust P Ami - The Chemist's Amanuensis J Cheminform 2011 3 45 10.1186/1758-2946-3-45 PMC3206454 21999587

